# Reconceptualizing Integrated Knowledge Translation goals: a case study on basic and clinical science investigating the causes and consequences of food allergy

**DOI:** 10.1186/s43058-023-00473-9

**Published:** 2023-09-27

**Authors:** Jenna Dixon, Emily Shantz, Ann E. Clarke, Susan J. Elliott

**Affiliations:** 1 Faculty of Health and Social Development, University of British Columbia (Okanagan), Kelowna, BC Canada; 2 Geography and Environmental Management, University of Waterloo, Waterloo, ON Canada; 3 Department of Medicine, University of Calgary, Calgary, AB Canada

**Keywords:** Knowledge Translation, Integrated Knowledge Translation, Implementation, Research use, Research co-production, Collaborative research, Food allergy, Knowledge-user

## Abstract

**Background:**

Integrated Knowledge Translation (IKT) and other forms of research co-production are increasingly recognized as valuable approaches to knowledge creation as a way to better facilitate the implementation of scientific findings. However, the nature of some scientific work can preclude direct knowledge to action as a likely outcome. Do IKT approaches have value in such cases?

**Methods:**

This study used a qualitative case study approach to better understand the function of IKT in a non-traditional application: basic and clinical science investigating the causes and consequences of food allergy. Building off previous baseline findings, data were obtained through in-depth interviews with project scientists and steering committee members and complemented by researcher observation. Data were analyzed through an integrated approach to understand how well participants perceived the stipulated project IKT outcomes had been met and to better understand the relationship between different forms of IKT goals, outcomes, and impacts.

**Results:**

We propose a conceptual model which builds temporal continuity into the IKT work and understands success beyond truncated timelines of any one project. The model proposes project IKT goals be conceptualized through three metaphorical tower blocks: foundational (changing the culture for both scientists and knowledge-users), laying the groundwork (building relationships, networks and sparking scientific inquiry), and putting scientific knowledge to action. Based on this model, this case study demonstrated notable success at the foundational and intermediate blocks, though did not turn basic and clinical research knowledge into actionable outcomes within the project timespan.

**Conclusions:**

We find that current IKT literature which situates success as filling a knowledge to action gap is conceptually inadequate for understanding the full contributions of IKT activities. This work highlights the need for building cultural and scientific familiarity with IKT in order to better enable knowledge to action translation. Improving understanding and communication of science and empowering knowledge-users to engage with the research agenda are long-term strategies to build towards knowledge implementation and lay the ground work for many future research projects.

**Supplementary Information:**

The online version contains supplementary material available at 10.1186/s43058-023-00473-9.

Contributions to the literature

- While Integrated Knowledge Translation/co-production approaches to research may address some barriers to implementation, the nature of some scientific work can preclude direct knowledge to action as a likely outcome.
- Current literature poorly theorizes how multiple research projects may build towards communal translation goals.
- Grounded in a case study investigating the causes and consequences of food allergy, we provide a conceptual model which theorizes Integrated Knowledge Translation outcomes and impacts beyond the domain of a single project.
- Integrated knowledge translation work should recognize changing the scientific culture as a first, not last, step in facilitating implementation.

## Background

Integrated Knowledge Translation (IKT) is one form of co-production where knowledge creation is a collaborative effort between researchers and users who “have the authority to implement the research recommendations” [1]; it is action-oriented and solutions-focused [2]. IKT, therefore, addresses insufficient implementation of research findings by recognizing that how we conduct science shapes subsequent uptake and impact [3]—both in steering research to relevant issues and in priming knowledge-users to understand and incorporate findings in their own work [4–6].

IKT can be more challenging as researchers navigate the intricacies of partnership building and relationship management [7, 8], and there is need to better understand outcomes and impacts [9, 10], what Kothari and Wathen see as the next major frontier in IKT science [11]. One of the challenges to this may be poor articulation in the literature between the goals of IKT (that is, the predefined purpose of enacting IKT, see Table 1), the conditions that drive success, and the outcomes and impacts that emerge. Hence, the literature calls for more theoretically informed approaches to IKT [11, 12].

**Table 1:** Definitions

Term	Definition
Outputs	The direct products/deliverables resulting from research activities. Outputs lead to outcomes. Includes reports, publications, presentations, communication strategies, education and training strategies, relationship building strategies and more.
Outcomes	Changes resulting from research activities and outputs; can include short-term, intermediate, and longer-term outcomes. Outcomes are measurable.
Impacts	Often used synonymously with outcomes, or as a collective term to encompass outputs, uses and outcomes (e.g., [13]). Here, we define as a futuristic identifiable benefit to, or positive influence on, society and other domains [14].
Goals	The predefined target outcomes and impacts of the research activities
Knowledge Translation (KT)	A dynamic and iterative process that includes synthesis, dissemination, exchange, and ethically sound application of knowledge. This process takes place within a complex system of interactions between researchers and knowledge-users which may vary in intensity, complexity, and level of engagement depending on the nature of the research and the findings as well as the needs of the particular knowledge-user. Usually framed as either end-of-grant or integrated [15, 16].
Integrated Knowledge Translation (IKT)	One form of research co-production which emphasizes the engagement of knowledge-users in research. Ideally, this engagement occurs for the entire research process including determining research questions, methodology, data collection, and tools development, interpreting the findings, and helping disseminate the research results [17, 18]. IKT is action-oriented and solutions-focused [2].
Research co-production	A broad definition of collaborative research where researchers work in partnership with knowledge-users. Encompasses terms such as participatory research, engaged scholarship, Mode 2 of knowledge production, collaborative research, or integrated knowledge translation (IKT) [19].

A range of perspectives is used to understand outcomes and impacts of IKT, when reported [20]. Reflecting IKT’s origins, outcomes often are discussed as immediate knowledge to action [10, 21] or Knowledge Translation (KT) products [8]. But research has also identified a variety of less tangible yet equally important outcomes such as tacit knowledge, social or relational capital, and the approach as a means of building relationships between knowledge communities [11, 22]. A recent umbrella review on research partnerships sorts outcomes and impacts by means of scale, such as individual level (either researcher scientists or stakeholders), the partnership-level (relationships between scientists and stakeholders), community level, or the research process more wholistically [14]. Beckett and colleagues likewise classify outcomes/impacts at various scales (individual, group, organizational, societal) but additionally consider “paradigmatic” contributions in which outcomes/impacts completely shift how the world is understood, what is legitimate knowledge, and the relationships between knowledge, research, and practice/policy [13]. This parallels Kothari and Wathen’s description of appreciating other points of view to converge on a newly created shared perspective [11]. The whole becomes more than the sum of its parts.

Many identified IKT outcomes hint at future programs of work without explicitly conceptualizing that process. For example, Sibbald and colleagues describe “for many researchers and knowledge-users, the impact was more about laying the groundwork for future research” [9]. On the other hand, many of the characteristics associated with successful IKT partnerships hint at previous IKT work without making this connection explicit—e.g., partnerships built on existing relationships, or the participation of skilled researchers (experienced in IKT) [23]. And, generally, co-production narratives recognize that partnership relationships that start from scratch require significantly more time investments in the early stages for learning and training, developing relationships, building trust, etc., and this may be a barrier to success [24]. Altogether, this creates a landscape in which the detailed inputs, outcomes, and impacts of IKT are documented but little is conceptually or theoretically understood about their relationship of these components to each other, especially beyond the timeline of a single project. Table 1 provides an overview of important terms used to inform this work.

Where many research projects may use IKT approaches for clear knowledge to action outcomes, the pertinency of these IKT goals in other forms of health research is less clear. As explored elsewhere [25, 26], the nature of some scientific investigations (e.g., basic, laboratory, discovery research) are slow and incremental and can preclude direct knowledge to action as a likely outcome—and hence, measure of success. This paper responds to that gap in the literature through a case study of a notably unorthodox application of IKT—that is, used in a grouping of basic and clinical science sub-studies investigating the causes and consequences of food allergy. Our purposes here are to (1) explore the IKT outcomes of this case and (2) understand these findings within broader conceptualizations of IKT success.

### Case study: IKT in the GET-FACTS project

**Fig. 1:**
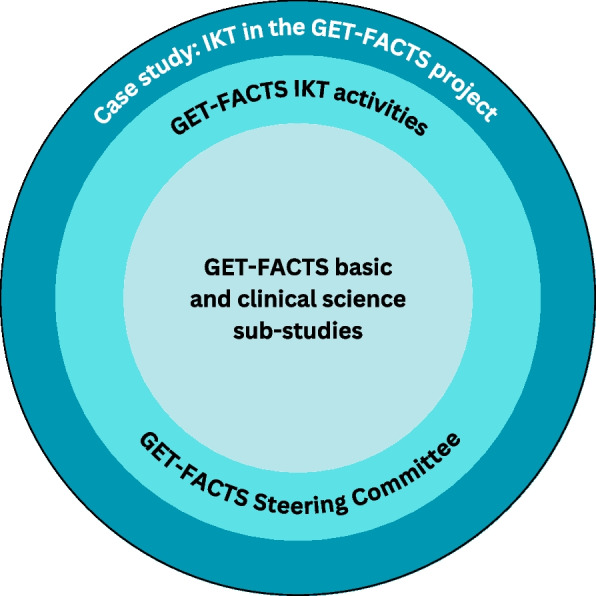
The case study: IKT in the GET-FACTS project

This is a case study on the IKT “experiment” through the GET-FACTS project, a 5-year biomedical research project funded by the Canadian Institutes of Health Research and led by a coalition of basic and clinical scientists to assess the causes and consequences of food allergy. As represented in Fig. 1, the case study’s focus was on the wholistic IKT experiences of the GET-FACTS project, inclusive of the core biomedical research components alongside the IKT activities (e.g., creation of the steering committee, setting target IKT outcomes). The timeline of the case study therefore parallels the duration of the GET-FACTS research activities.

Food allergies are a potentially life-threatening chronic health issue and public health concern [27], with significant impacts on affected individuals, their families, social and educational settings, and communities, as well as the healthcare system more broadly [28]. Within the global food allergy community, there was pressing need to better connect knowledge-users with the science, after standard clinical recommendations for allergy prone children to delay introduction of potential allergenic foods, such as peanuts, were turned upside down. Though there were hints that previous recommendations were misguided [29], a landmark study in 2015 found that infants with delayed peanut introduction were actually at greater risk of developing food allergies—counter to previous understanding [30]. Clinicians and knowledge-user organizations worldwide scrambled to update and communicate new recommendations while assuring parents they had acted appropriately given the body of knowledge at the time [31–34]. There was recognition from all parties on the importance of knowledge-users being closer to the science and for scientists to be able to better communicate their processes to knowledge-users.

Enter the GET-FACTS project; originally conceived as a united but distinct collection of basic and clinical sub-studies within three major pillars of investigation (genetic determinants of food allergy and tolerance, environmental impact on functional and immunological tolerance to foods, and novel biomarkers to assess allergy and tolerance), co-investigators identified the need for active involvement of knowledge-users to ensure the usability of the research. As such, social scientists with expertise in knowledge translation joined the research team and a knowledge-user driven IKT agenda was built into the protocol to work on behalf of and alongside the basic and clinical researchers towards achieving IKT goals. A knowledge-user steering committee was created to learn about GET-FACTS research as it was unfolding, assist in interpretation, and identify IKT opportunities and potential outcomes associated with the project. Six representatives from Canadian food allergy policy and advocacy participated in drafting the project proposal and, after it was successful, additional members were invited to ensure comprehensive representation. In total, eight knowledge-users sat on the steering committee representing organizations involved in food allergy information and advocacy, Canadian health and wellbeing information and advocacy, science policy brokering, government, and public health. Three steering committee members additionally brought personal experiences navigating their own or an immediate family member’s anaphylactic food allergy.

As part of the case study investigation, year-1 qualitative interviews were conducted with all basic and clinical scientists and steering committee members to better understand the base level of knowledge regarding IKT and expectations for the project work. While not the focus of this paper, the results, presented in-depth elsewhere [35, 36], provide important context for the direction of IKT activities. Baseline assessments demonstrated optimism from both scientists and steering committee members on the potential outcomes from this IKT application; however, a number of flags emerged. Scientists on the project were aware of the term Knowledge Translation (KT) but primarily emphasized the activity as “bench to bedside” clinical applications or end-of-grant dissemination activities to the public, having no “integrated” research experiences to draw on. Where project clinicians had experience working with non-researchers (e.g., patients in clinic, patients enrolled in studies), experience among basic scientists was notably limited, to which one participant reflected, “we've never received any formal training in that area” [35]. In the context of the IKT, a number of scientists expressed concern about findings being translated too soon without proper contextualization, the relatively slow pace of scientific advancement (particularly so for basic science), and the public’s need for quick and immediate answers. Steering committee members, in contrast, had experience with science-policy bridging but raised concerns that many months into the project they did not feel knowledgeable or connected to science and asked for more regular touch points between the scientists and steering committee. Notably, steering committee members envisioned success (creating a concrete resource for others to draw on, replication of this IKT approach in future research projects) and, conversely, failure (if approach did not influence the conduct of future research projects) through very future-focused terms [36].

A number of actions were taken in response to this feedback. More touchpoints were created beyond the once or twice annual all-researcher meeting and presentations. Senior scientists representing the various GET-FACTS sub-studies led webinars with time for discussion with the steering committee. This aimed to strengthen steering committee members’ familiarity with the project’s scientific activities, improve communication, and better build relationships. In addition to the steering committee created Terms of Reference, which included project outcomes/impacts of interest, an exhaustive process was undertaken with project members (both scientists and steering committee) to better articulate their IKT goals, and operationalize activities, outputs, and outcomes through a Performance Measurement Framework (PMF).

The completed PMF outlined three streams (communication and education, networking and relationship building, evaluation and accountability) of activities and outputs to inform envisioned short-term, intermediate and long-term outcomes. The five PMF articulated short-term target outcomes were as follows: (1) steering committee members have a greater awareness, understanding and knowledge about research, (2) steering committee members feel more empowered to contribute to the research process, (3) scientists have increased awareness, understanding and knowledge about IKT, (4) scientists feel more empowered to contribute to the IKT process, and (5) relationships are strengthened between project scientist and steering committee members. The sole PMF articulated intermediate target outcome was the creation of an IKT approach for replication in similar types of research. The three PMF articulated long-term target outcomes were (1) evidence informed policy and decision making, (2) future scientific research is shaped by end users in collaboration with scientists, and (3) maximize choice and minimize risk for Canadians affected by food allergies. Through this process, the steering committee and research team reflected that these target outcomes did not align with common IKT action-focused outcomes (e.g., outcomes paid little attention to specific knowledge products, or specific changes to food allergy policy and practice) and instead articulated a program of work which focused on the nature of research and building relationships between knowledge-creators and knowledge-users.

## Methods

This was a qualitative case study [37, 38] of the GET-FACTS research project, with the objective to better understand the function of IKT approaches within basic and clinical biomedical research. A case study “investigates a contemporary phenomenon within its real-life context and addresses a situation in which the boundaries between phenomenon and context are not clearly evident” [39] and is valuable for capturing that complexity and providing in-depth understanding of phenomena [38, 40]. In line with Stake [41] and Merriam’s [42] social constructivist approach to case studies, the researcher may also have a personal interaction with the case and the context of the case may shift over time, as was relevant in this current study. We reported the study using the COREQ (Consolidated criteria for reporting qualitative research) Checklist [43] (Additional file 1).

### Data collection and analysis

As noted above, baseline in-depth interviews conducted year-1 with all core GET-FACTS scientists and steering committee members have been reported [35, 36], as have early reflections on the challenges of doing IKT in basic and clinical research contexts [25]. These early insights provide important framing for this summative report. Data here have been generated from semi-structured in-depth interviews conducted at the conclusion of the project with all (100% response) twelve core GET-FACTS basic and clinical scientists and eight steering committee members. Participants were made aware at in-person project meetings of the opportunities for concluding interviews and were later contacted by ES through email with detailed information. Interview questions queried awareness, understanding, knowledge about research and IKT, perceived overall project outcomes, and nature of relationships between researchers and knowledge users over the life of the project. As external to the project, ES, a female PhD student with a master’s degree being trained by SJE on qualitative interview techniques, conducted the interviews to minimize positive response bias. Interviews averaged around 50 min, were conducted over the phone, recorded, and later transcribed verbatim. SJE, AEC, and JD participated in GET-FACTS IKT activities and research meetings; AEC is also a core GET-FACTS clinical research scientist. SJE, AEC, and JD therefore also contribute to the case study analysis with embedded researcher observations. Research ethics board approval was received prior to all research activities (University of Waterloo ORE# 19735).

We adopted an integrated approach for the analysis [44], which was completed in a two-part process. First, in order to understand how well participants perceived the IKT target outcomes had been met, we created a coding framework which encompassed the same coding as in baseline qualitative interviews and also included coding relevant to an assessment of the project outcomes as had been developed mid-project. The coding, therefore, was largely deductive (based on a pre-established coding framework) but sub-codes that emerged inductively were incorporated into the analysis [44]. ES coded each transcript in full, verified for reliability with JD, as overseen by SJE.

The second part of analysis sought to conceptualize the relationship between different forms of IKT goals, outcomes and impacts. For this, we enacted an iterative analysis as a back-and-forth conversation [45] between the qualitative data, the literature, and our experiences as researchers working in IKT research broadly and the GET-FACTS IKT case study specifically. Iterative analyses embrace the researcher’s reflexivity as an important part of the process [46, 47], acknowledging the interaction between what the “data are telling me” and “what I want to know” [46]. Qualitative transcripts were cleared of previous analysis and coded openly for any references to outcomes and impacts as perceived by participants (conceptual codes), the links between these codes (relationship codes), and any direction that was associated with these links (participant perspective) [44]. JD completed all initial coding, verified for reliability with ES, as overseen by SJE. Codes, and the relationships between them, were sorted and discussed by the research team in context of the IKT and co-production literature. Though the first-phase coding was “removed,” the findings informed the views of the JD, SJE, and ES in discussion of broader theorizing on IKT outcomes as part of the iterative “back and forth” reflection. The research team finalized the model as a simple but productive way to conceptualize IKT outcomes, which reflect a trifecta of the data, our research experiences, and the broader literature. As a final step in this process, the findings from phase 1 of analysis were mapped onto the conceptualization borne out of phase 2 of analysis. The results of this conceptualization are therefore presented first, and then used to structure findings on the GET-FACTS target outcomes.

## Results

### The Tower Model

The need for a reframed thinking was evident through the GET-FACTS project both through what incoming challenges were faced in year 1 (e.g., a lack of scientific cultural frameworks for incorporating co-production, poor understanding of the rigor of IKT science) and how participants constructed outcomes and impacts at the end of project. We identified as a gap in the literature the need for better understanding of how one project can support IKT co-production in subsequent projects. We noted the words “build” or “building” emerged frequently in the interviews, used by all but one steering committee member and two scientists. Conceptually, participants described some IKT outcomes as building upon other IKT outcomes, and project goals are best achieved when all outcomes are done well and build “up” activities over time. This informed the visual metaphor for our model as a tower of building blocks.

**Fig. 2:**
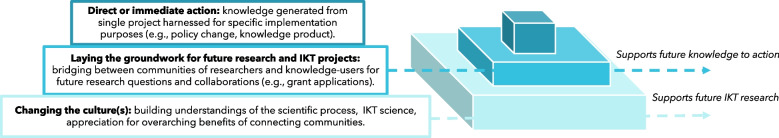
The Tower Model for conceptualizing goals of IKT research

The conceptual model (Fig. 2) identifies three distinct levels or “blocks” of success created through IKT work. It recognizes success stemming from one project as building blocks towards success in future projects, creating a temporal continuity that has been poorly conceived of to date. The foundational block, titled “changing the culture,” includes outcomes/impacts that build understandings of the scientific process, legitimizes IKT science, and validates connection and collaboration with/between scientists and knowledge-users as a fundamental aspect of research. It also represents “paradigmatic” shifts [13] in which outcomes/impacts shift how the world is understood, what is legitimate knowledge, and the relationships between knowledge, research, and practice/policy. This can occur at multiple scales from individual to institutional. The intermediate block(s) build off the former with more targeted applications. Titled “laying the groundwork,” this includes outcomes that enable future research and IKT projects. This can include the building of relationships and collaborations between scientists and knowledge-users in specific contexts (e.g., food allergy scientists’ relationships with food allergy knowledge-user organizations) or the generation of research areas or questions to be explored in future projects. The upper block(s), titled “knowledge to action,” encapsulate more traditional KT outcomes and reflect knowledge to action from a single project such as knowledge tools, knowledge dissemination, or change to policy or clinical practice and additionally includes the influence that knowledge-users have on the science of that project (e.g., patient recruitment, reframed interpretation of findings). Any block or multiple blocks may be the IKT goal (targeted outcome) for a project but building strength through the foundation up enables not only actionable outcomes from a single project but also supports building further with future projects.

### IKT in the GET-FACT project: target and realized IKT outcomes

GET-FACTS scientists and steering committee members participated in an extensive exercise to articulate a joint vision for IKT activities, which resulted in five target short-term outcomes. The realization of these outcomes was gauged through the case study’s concluding qualitative interviews. The intermediate and long-term outcomes envisioned by the steering committee and scientists have timelines beyond the scope of this case study, but as *goals* are relevant in connection to the Tower Model. Table 2 re-imagines these envisioned outcomes (short, intermediate and long term) in relation to the conceptual addition of the Tower Model. Table 2 demonstrates that GET-FACTS target IKT outcomes were primarily concerned with what the Tower Model classifies as foundational. Additionally, while there was also attention on the intermediate block, the top block of “knowledge to action” was only considered in relation to long-term outcomes.

**Table 2:** Connecting GET-FACTS IKT target outcomes to the Tower Model

GET-FACTS target outcomes	**Tower Model**
**Target short-term outcomes (goals)**	
Steering committee members have greater awareness, understanding, and knowledge about research	Foundational block, “changing the culture”
Steering committee members feeling more empowered to contribute to the scientific process	Foundational block, “changing the culture”
Scientists have increased awareness, understanding, and knowledge about integrated Knowledge Translation	Foundational block, “changing the culture”
Scientists feel more empowered to contribute to the integrated knowledge translation process	Foundational block, “changing the culture”
Strengthened relationships between GET-FACTS project scientists and steering committee members	Intermediate block, “laying the groundwork”
**Target intermediate outcome (goal)**	
Creation of detailed IKT approach	Intermediate block, “laying the groundwork” and top block, “knowledge to action”
**Target long-term outcomes (goals)**	
Evidence informed policy and decision making	Top block, “knowledge to action”
Future scientific research is shaped by end users in collaboration with scientists	Intermediate block, “laying the groundwork” and foundational block, “changing the culture”
Maximize choice and minimize risk for Canadians affected by food allergies	Top block, “knowledge to action”

An overview of perceived study outcomes as assessed through the qualitative interviews with project scientists and steering committee members is presented in Table 3. Results described here are structured by the three tower blocks and illustrated with quotations from scientist (Sci) and steering committee (SC) participants.

**Table 3:** GET-FACTS IKT outcomes

End-of-project: findings from concluding case study interviews with GET-FACTS scientists (number of participants who referenced/12 total)	End-of-project: findings from concluding case-study interviews with GET-FACTS steering committee members (number of participants who referenced/8 total)
1) Knowledge to action	
♦ Scientists describe IKT as having positive impacts on dissemination of research findings, including: networks enhance dissemination (3/12), research is more useful and applicable (3/12), and information is disseminated more accurately (1/12) ♦ Limited number of scientists identify change in the research focus (1/12) and process (2/12) from connecting with steering committee. Reasons included: too early to tell, feedback was positive, nature of study cannot be changed ♦ Limited number of scientists identify change in KT practices from steering committee connections (2/12) and no (0/12) scientists identify improved access to resources such as patients or samples	♦ Steering committee members describe IKT as impacting the eventual dissemination of GET-FACTS research (knowledge-users participate in dissemination process, dissemination is more relevant, new networks are reached with this information) (7/8) but describe it being too soon for dissemination of research information (2/3) ♦ Steering committee members describe participation in IKT process as making them more confident in educating others (4/8) ♦ Steering committee members describe having a better understanding of the scientific process (5/8) ♦ Steering committee members describe IKT as having an impact on the general work produced through the project (4/8), with some specifically noting an impact on the work produced by scientists as informed by knowledge-users (3/8) and more relevant/meaningful (2/8) ♦ Steering committee members identify knowledge-users as having informed the GET-FACTS research process (3/8) and some identify this research as more meaningful/relevant because of this (2/8)
2) Laying the groundwork	
♦ Scientists reference relationships built between scientists and knowledge-users as a marker of success from the project (8/12) ♦ Scientists identified either new (3/12) or strengthened (4/12) relationships with knowledge-user organizations ♦ Scientists identify steering committee relationships as motivating or shaping interests in the research area (4/12) ♦ Scientists are able to describe the GET-FACTS IKT approach with a lot (4/12) or some (4/12) familiarity and detail ♦ No scientists (0/12) identified at the time of interview having next research opportunities or collaborations stemming from work with steering committee	♦ All steering committee members said they either formed new relationships (5/8) and/or strengthened existing relationships (4/8) among knowledge-user organizations ♦ Steering committee members are able to describe the GET-FACTS IKT approach with a lot (4/8) or some (4/8) familiarity and detail ♦ Steering committee members describe utility of IKT approaches generally or PMF specifically for other work they are doing (3/8)
3) Changing the culture	
♦ All scientists (12/12) are able to critically describe advantages and challenges to doing IKT work. Advantages most cited: integrates multiple perspective (9/12), knowledge-users receive more accurate information (4/12), research is more meaningful to knowledge-users (4/12), knowledge-users are aware of the research process (3/12). Challenges most cited: difficult to integrate multiple perspectives (6/12), required time investment (4/12), challenge in communicating science (3/12) ♦ Scientists (7/12) identify this project as changing their knowledge or understanding of KT. This is supported by analysis of questions on KT (e.g., whose job is it to do KT? What is the role of basic/clinical scientists in KT?). Compared to baseline interviews, scientists now place far greater emphasis on the role of scientists in KT (8/12), KT as a shared responsibility (7/12), the importance of two-way dialogue (6/12) ♦ Scientists (4/12) identify specific strategies for scientists and knowledge-users to work together which reflect the literature on IKT best practices: e.g., involve knowledge-users from the very beginning (4/12), regular communication and engagement (4/12), include those experienced in IKT (2/12) ♦ Scientists reference a change in science practice broadly (4/12) and similar research being conducted using IKT (3/12) as markers of success from the project ♦ Scientists note their “biggest surprise” during the IKT process was learning the scientific validity of KT science (2/12)	♦ Steering committee members are all able to describe the GET-FACTS IKT activities (8/8) ♦ Steering committee members are all able to describe differences between end-of-grant and IKT approaches to KT, and are able to describe both advantages and challenges of doing IKT work (8/8) ♦ Steering committee members describe greater understanding of science (specifically science related to food allergy) compared to before the project (5/8), though some describe no change (2/8) ♦ Steering committee members articulate limitations of scientific research findings (broadly) with regards to their own work and discuss broad implications for how to build research into their organizations and communications strategies (5/8) ♦ Steering committee members describe similar research being done with IKT as marker of success from the project (4/8) ♦ Steering committee members note their “biggest surprise” during the IKT process was the willingness of scientists to participate (3/8)

### Changing the culture

Scientists self-described as having expanded their knowledge or understanding of IKT as a result of this project. Comparison with the case study’s baseline findings [35] supports evidence of this change. For instance, when asked broad questions about the larger concept of KT (e.g., whose job is it to do KT? What is the role of basic/clinical scientists in KT?), scientists originally emphasized other actors as being responsible for KT, whereas by the concluding interviews analyzed here, far greater emphasis was placed on the role of scientists in KT, KT as a shared responsibility, and the importance of two-way dialogue.

Scientists described a change perception in IKT as a rigorous and validated body of knowledge, and two even described their “biggest surprise” in this process was learning the scientific validity of IKT:

Well the entire program was new to me [laughs]! You know, when GET-FACTS started, IKT was kinda something that fluffy people did in corners and not really a hard science. And so I think that the team has done really well at changing perceptions and demonstrating the validity and value of that kind of reorientation of a research question. Which is really what it is. So that was a surprise to me. (Sci 1).

In contrast, steering committee members described familiarity with the rigor of IKT but many expressed surprise at the openness of the scientists in participating.

Scientists demonstrated fundamental shifts in their knowledge and understandings regarding KT broadly and IKT specifically. For instance, all scientists were able to critically describe advantages and challenges to doing IKT work. Advantages most cited were that it integrates multiple perspective, knowledge-users receive more accurate information, research is more meaningful to knowledge-users, and knowledge-users are aware of the research process. Challenges most cited were difficult to integrate multiple perspectives, required time investment, and challenge in communicating science. Additionally, scientists identified specific strategies for scientists and knowledge-users to work together which reflect the literature on IKT best practices, such as to involve knowledge-users from the very beginning, to regularly communicate and engage, and to include those experienced in IKT.

Though not as much of a shift from baseline data, steering committee members likewise articulated greater knowledge of IKT stemming from their involvement in the project. All were able to describe differences between end-of-grant and IKT approaches to KT and to describe both advantages and challenges of doing IKT work and to describe the GET-FACTS approach to IKT in detail.

My knowledge view and understanding of integrated knowledge translation has primarily been shaped by my involvement in the GET-FACTS project. I don’t think I had a clear appreciation of its value and of the difference between it and just knowledge translation before I started my involvement in this project. (SC 2).

In a similar vein, steering committee members, unprompted, articulated limitations of scientific research findings with regard to their own work and discussed broad implications for how to build research into their organizations and communications strategies:

[In regards to the study which upended food allergy recommendations [30]] Many, many of our parents are still very, very confused and we have to tell them that you know what, what you’re doing is fine. This is a very, very small test subject matter. There needs to be more studies done before it becomes conclusive. (SC 7).

Finally, where both scientists and steering committee members saw as an important marker of success from the project that similar research will be conducted using IKT, scientists additionally point to success as a change in science practice broadly:

Well I think firstly it sensitizes people about the fact that scientists think one way, people who are affected with a disease think another way, stakeholders and policy makers think a different way. If you don’t meet the minds, if people are not all talking to each other, you can’t be sensitive to how other people look at problems, how other people need information to solve problems, you know. It’s really crucial to not just think you know everything and not think you have all the answers. And I think that’s what, to me, this was the most valuable part of this whole exercise, to give you a little bit of humbleness and say, we don’t have everything, we don’t know all the issues that go into a family or policy maker or group that is trying to sensitize families to best practices, to educate families. How are we going to really build an ecosystem of health together? And this, I think, was the most valuable message of the integrated KT project that we’ve put into place. (Sci 10).

### Laying the groundwork

Both scientists and steering committee members described new and/or strengthened relationships with knowledge-user organizations because of their involvement in GET-FACTS. While the majority of scientists described the building of these relationships as a marker of success from the project, none had specifically identified next research opportunities stemming from these relationships.

Interactions with knowledge-users on the project influenced thinking and directions future research projects will take:

So I mean, I can’t say I walked out of a GET-FACTS meeting and into the lab and did a new experiment. It’s not that direct a line, but it’s the generation of ideas that sort of gets you thinking in other directions… (Sci 8).

And one steering committee member recalled a moment where very evidently the collaboration between scientists and knowledge-users sparked new directions:

I don’t know how the scientists feel but, from my point of view, I mean we had a magical moment back [at the last researcher meeting]. We had all these scientists in a room who had done presentations for us and we got to know a little bit over the years and who are just amazing people. They for the first time ever, they all had a thought at the same time that there was something that they could be working on, a research root that they should be working on. And they could make it happen because there was some sort of application due right away and somebody ran off to fill in the application so they wouldn’t miss the timeline. And for a brief moment all of us were on the same page, working on a common issue. And for me, that’s…. that was a magical moment. (SC 1).

A number of scientists described specific intent to adapt the GET-FACTS approach to IKT into upcoming projects that were in formation:

I’m actually going to implement this in a large project that we put forward. I think right from the beginning of the project you have to embrace the concept that you need end users and people who are non-scientists in the planning and in the implementation process of your grants. Knowledge translation is not just publishing a paper or going to a conference where there’s a bunch of medical personnel who are going to listen to and you know, you’re preaching to the converted. Knowledge translation really is all about making sure that there is a concerted effort right from the beginning of any project to have your stakeholders on board, especially if it’s a large clinical project. I mean, perhaps some basic science studies don’t need this, but we certainly learned our lesson and I’m implementing that in another large clinical setting that I’m working on now. (Sci 10).

Steering committee members similarly expressed interest in adapting the GET-FACTS approach to IKT towards future projects:

I actually have a note to call [IKT lead] and ask if I can share the framework with some of my [colleagues]…it’s a big national project that’s going underway and they desperately need some integrated knowledge mobilization work. (SC 5).

Finally, most of the scientists and steering committee members were able to describe the GET-FACT approach to IKT with specificity, suggesting their knowledge could be translated to future research projects.

### Knowledge to action

Overall, participants perceived low levels of direct action (e.g., dissemination, tools, changes to policy practice) that had emanated from the project through IKT process at the time of the interviews. As described in Table 2, the IKT PMF focused largely on foundational and intermediate tower blocks as outcomes and knowledge to action was not pinpointed as a short-term outcome goal (one exception here was the creation of the IKT PMF itself, as a tool for future research programs). This also reflected the unpublished status of much of the science in the project and the steering committee’s agreement to keep all information in confidence until approved for public use (peer-reviewed publication):

I haven’t seen any of the output from any of the folks other than what they presented to us which was still work in progress. (SC 1).

However, a majority of the scientists and steering committee members described optimism that the IKT approach would have positive impacts on dissemination at the appropriate time.

A limited number of scientists identified change in their research focus or process, and none identified improved access to resources such as patients or samples, though there were discussions of patient recruitment for genetic analysis at the mid-point of the project. However, steering committee members described having subtle impacts on the framing and analysis of research findings through their interactions and sharing world views:

Questions that the stakeholders or the steering committee members asked the researchers is a different angle or different interpretation of the results that the researchers hadn’t necessarily thought of. (SC 8).

Finally, one scientist expressed disappointment at the focus of IKT activities:

I didn’t realize [the intent] was just to conduct kind of a conceptual framework and to kind of have this be an esoteric project. I had thought that it was actually meant to really have that kind of impact and to actually have that translation to the actual research teams. And, I don’t think that was the intent, I think I had misunderstood. And if it was the intent, that unequivocally was a huge fail… (Sci 5).

This sentiment underscores the need to ensure expectations are clearly articulated and communicated among all participants in IKT projects.

## Discussion

Changing the culture is a marathon not a sprint; not only that, it is foundational to IKT [25]. The GET-FACTS project scientists and steering committee members together decided to put culture change at the forefront of the agenda. Over the course this 5-year case study, significant change occurred in how research scientists understand knowledge and IKT co-production and how knowledge-users understand science itself and their role in it. For example, where baseline data revealed many scientists having dismissive or skeptical attitudes towards the engagement of knowledge-users in the research process, concluding interviews revealed a transformation in the attitudes of scientists towards such engagement. This change marks a dramatic epistemological shift away from linear positivistic understandings of knowledge towards the more complex and interpretive epistemological foundations of co-creation. While there is certainly more work to be done for broader change in the norms of scientific culture, the shifts here align with what Beckett et al. [13] describe as paradigmatic.

Less success was evident in moving knowledge into action (Tower Model’s top block). This reflects the convergence of many factors, including the slow incremental nature of basic science, the lack of relationships between scientists and knowledge-users at the onset of the project, and the need to prioritize foundational/cultural change as the primary focus of efforts. While the vast majority of feedback from scientists and knowledge-users was supportive of the IKT activities, this was not universal, and there was disappointment expressed from one scientist on the lack of knowledge products emerging from IKT activities. Further conversation on the purpose and measures of success in IKT is necessary. The IKT literature values the tacit outcomes of co-production [11, 15] but, ultimately, scientists, knowledge-users and funders expect the time and expense of partnering to be *for* something; this may be challenging to demonstrate over short timeframes.

IKT goals (target outcomes) will vary for every project. The Tower Model is conceptualized to help researchers reflect on these goals to ask: where are you going and why? If the goal is meaningful and actionable change to policy and practice, do you have the foundation in place to facilitate that change? Such considerations draw from the insights of many other approaches to co-production which have clearly demonstrated the vital importance of building projects on foundations of trust [8, 16, 48–50]. The Tower Model, however, aligns with IKT practices of prioritizing the knowledge to action outcomes as important, albeit here through an extended timeline. Further, complementary to existing work, the model focuses on bringing research to a point where planned action theories, such as the Knowledge to Action (KTA) framework, may be used to enhance implementation efforts [51, 52].

Researchers who are trained to think about IKT success through multiple projects over the course of time may spend early-years energy focusing on the lower foundational and groundwork blocks in order to build the capacity for meaningful action in later projects. Knowing the challenging and time-consuming nature of this type of work [12], researchers do themselves a disservice to have to start from scratch every time. There is also a risk of falling victim to what Kothari and Wathen [15] call the positivity bias in IKT—that is, where scientists and knowledge-users enter co-production partnerships assuming there will be definitive evidence on a specific problem and they will together generate solutions that work. This does not reflect the messy and sometimes disappointing and inconclusive reality of doing of research. It is therefore imperative that IKT work recognizes success in many forms, as building blocks to future research and future implementation efforts.

Where agencies fund in short increments (1, 2, or, as in this case, 5 years), strong foundations and partnerships are established just as project timelines conclude. Evidence suggests that the periods between funded research may strain co-production relationships [50, 53], as resources are understood as critical to maintain strong researcher-knowledge user partnerships [54]. In many cases, meaningful solutions-focused outcomes would be best supported over extended research timelines, and funders should consider this in program implementation. For instance, Centre of Excellence (CoE) schemes, which already focus energy towards knowledge and competence building and cross community transfer [55], could be harnessed to simultaneously build IKT specific expectations over the long-term (e.g., years 1–5 a focus on foundational cultural change, years 6–10 a focus on strong partnership and networking, years 10 + a focus on relevant knowledge products).

There are a few of limitations of this work that bear highlighting. First, there are many good reasons to engage in knowledge co-production, but the Tower Model reflects the IKT emphasis on being action oriented and solutions focused and filling the knowledge to action gap. Not all co-production projects will find this as the north star and this should be interpreted appropriately. Second, despite efforts to minimize positive response bias, we must acknowledge this may be at play in some instances. Finally, though situated within the broader literature, the Tower Model is primarily based on one central IKT project. Further testing and discussion of the model are welcome and encouraged.

## Conclusions

In the wake of revelations on the causes of food allergy [30], a disconnect appeared between the nature of incremental research science and the need for public policy to ensure health and safety for those with food allergy. In this context, the GET-FACTS IKT project sought to maximize the impact of its research through deep change in how scientists and knowledge-users understand each other’s worldviews. Improving understanding and communication of science and empowering knowledge-users to engage with the research agenda is a long-term strategy to build towards knowledge implementation. Activities focused on these goals build a foundation and lay ground work for many future basic and clinical research projects, with tremendous potential to ripple onward into meaningful policy and practice.

### Supplementary Information

**Additional file 1.** 

## Data Availability

The dataset analyzed during the current study is not publicly available due to the protection of the human subjects involved in the qualitative interviews. A deidentified copy of the dataset is available from the corresponding author on reasonable request.

## Abbreviations

IKT: Integrated Knowledge Translation
KT: Knowledge Translation
PMF: Performance Measurement Framework

## References

[1] Kothari A, McCutcheon C, Graham ID (2017). Defining integrated knowledge translation and moving forward: a response to recent commentaries. Int J Health Policy Manag.

[2] Bowen SJ, Graham ID (2013). From knowledge translation to engaged scholarship: promoting research relevance and utilization. Arch Phys Med Rehabil.

[3] Bowen S, Botting I, Graham ID, Huebner LA (2016). Beyond “two cultures”: guidance for establishing effective researcher/health system partnerships. Int J Health Policy Manag.

[4] Cardwell FS, Elliott SJ, Clarke AE (2021). The value of hackathons in integrated knowledge translation (iKT) research: Waterlupus. Health Res Policy Syst.

[5] Bucknall T (2012). Bridging the know-do gap in health care through integrated knowledge translation. Worldviews Evid Based Nurs.

[6] Graham ID, Kothari A, McCutcheon C (2018). Moving knowledge into action for more effective practice, programmes and policy: protocol for a research programme on integrated knowledge translation. Implement Sci.

[7] Rycroft-Malone J, Burton CR, Bucknall T, Graham ID, Hutchinson AM (2016). Collaboration and co-production of knowledge in healthcare: opportunities and challenges. Int J Health Policy Manag.

[8] Cooke J, Langley J, Wolstenholme D, Hampshaw S (2016). Seeing” the difference: the importance of visibility and action as a mark of “authenticity” in co-production: comment on “Collaboration and co-production of knowledge in healthcare: opportunities and challenges. Int J Health Policy Manag.

[9] Sibbald SL, Kang H, Graham ID. Collaborative health research partnerships: a survey of researcher and knowledge-user attitudes and perceptions. Health Res Policy Syst. 2019;17(1):1–10. 10.1186/s12961-019-0485-3 PMC6880346 31775829

[10] Kreindler SA (2018). Advancing the evaluation of integrated knowledge translation. Health Res Policy Syst.

[11] Kothari A, Wathen CN. Integrated knowledge translation: digging deeper, moving forward. J Epidemiol Community Health (1978). 2017;71(6):619–23. 10.1136/jech-2016-208490 28298415

[12] Gagliardi AR, Berta W, Kothari A, Boyko J, Urquhart R (2015). Integrated knowledge translation (IKT) in health care: a scoping review. Implement Sci.

[13] Beckett K, Farr M, Kothari A, Wye L, le May A (2018). Embracing complexity and uncertainty to create impact: exploring the processes and transformative potential of co-produced research through development of a social impact model. Health Res Policy Syst.

[14] Hoekstra F, Mrklas KJ, Khan M, McKay RC, Vis-Dunbar M, Sibley KM, et al. A review of reviews on principles, strategies, outcomes and impacts of research partnerships approaches: a first step in synthesising the research partnership literature. Vol. 18, Health Research Policy and Systems. BioMed Central Ltd.; 2020. 10.1186/s12961-020-0544-9 PMC7249434 32450919

[15] Kothari A, Wathen CN (2013). A critical second look at integrated knowledge translation. Health Policy (New York).

[16] Culpin I, Dermott E, Ives J, MacLeavy J (2021). Tangible co-production?. Engaging and creating with fathers Area.

[17] McLean RKD, Graham ID, Bosompra K, Choudhry Y, Coen SE, MacLeod M (2012). Understanding the performance and impact of public knowledge translation funding interventions: protocol for an evaluation of Canadian Institutes of Health Research knowledge translation funding programs. Implement Sci.

[18] Nguyen T, Graham ID, Mrklas KJ, Bowen S, Cargo M, Estabrooks CA (2020). How does integrated knowledge translation (IKT) compare to other collaborative research approaches to generating and translating knowledge? Learning from experts in the field. Health Res Policy Syst.

[19] Graham ID, McCutcheon C, Kothari A (2019). Exploring the frontiers of research co-production: the Integrated Knowledge Translation Research Network concept papers. Health Res Policy Syst.

[20] Camden C, Shikako-Thomas K, Nguyen T, Graham E, Thomas A, Sprung J (2015). Engaging stakeholders in rehabilitation research: a scoping review of strategies used in partnerships and evaluation of impacts. Disabil Rehabil.

[21] Bird ML, Mortenson BW, Chu F, Acerra N, Bagnall E, Wright A (2019). Building a bridge to the community: an integrated knowledge translation approach to improving participation in community-based exercise for people after stroke. Phys Ther.

[22] Rycroft-Malone J, Wilkinson J, Burton CR, Harvey G, McCormack B, Graham I, et al. Collaborative action around implementation in Collaborations for Leadership in Applied Health Research and Care: towards a programme theory. J Health Serv Res Policy. 2013;18(3_suppl):13–26. 10.1177/1355819613498859 24127357

[23] Sibbald SL, Tetroe J, Graham ID (2014). Research funder required research partnerships: a qualitative inquiry. Implement Sci.

[24] Zych MM, Berta WB, Gagliardi AR (2020). Conceptualising the initiation of researcher and research user partnerships: a meta-narrative review. Health Res Policy Syst.

[25] Dixon J, Elliott SJ (2019). Changing the culture is a marathon not a sprint. Allergy Asthma Clin Immunol.

[26] Van Olphen J, Ottoson J, Green L, Barlow J, Koblick K, Hiatt R (2009). Evaluation of a partnership approach to translating research on breast cancer and the environment. Prog Community Health Partnersh.

[27] Shantz E, Elliott SJ. Chronic disease. In: International Encyclopedia of Human Geography. 2nd ed. Netherlands: Elsevier; 2020. p. 187–91. ISBN (Electronic) 9780081022962.

[28] Golding MA, Simons E, Abrams EM, Gerdts J, Protudjer JLP (2021). The excess costs of childhood food allergy on Canadian families: a cross-sectional study. Allergy Asthma Clin Immunol.

[29] Wennergren G (2009). What if it is the other way around? Early introduction of peanut and fish seems to be better than avoidance. Acta Paediatr.

[30] Du Toit G, Roberts G, Sayre PH, Bahnson HT, Radulovic S, Santos AF (2015). Randomized trial of peanut consumption in infants at risk for peanut allergy. N Engl J Med.

[31] Greenhawt M, Stukus D. New peanut allergy study does not say parents are to blame kids with food allergies [Internet]. Kids with food allergies. 2015 [cited 2021 Dec 29]. Available from: https://community.kidswithfoodallergies.org/blog/new-peanut-allergy-study-does-not-say-parents-are-to-blame-1.

[32] Mazer B. Shunning all peanuts may do harm, not good; could giving infants peanuts lessen allergy risk? Studies suggest yes. Montreal Gazette. 2015;A.15-A.15.

[33] Allergic Living. What LEAP study’s results mean for you. Allergic Living [Internet]. 2015 Mar 19 [cited 2021 Dec 29]; Available from: https://www.allergicliving.com/2015/03/19/what-leap-means-to-your-family/.

[34] Fleischer DM, Sicherer S, Greenhawt M, Campbell D, Chan E, Muraro A (2015). Consensus communication on early peanut introduction and the prevention of peanut allergy in high-risk infants. J Allergy Clin Immunol.

[35] Dixon J, Elliott SJ, Clarke AnnE. The co-production of biomedical research in Canada: are scientists ready to take the plunge? An Empirical Example from Food Allergy Research. Univers J Public Health. 2017;5(5):197–205.

[36] Dixon J, Elliott SJ, Clarke AE. Exploring knowledge-user experiences in integrated knowledge translation: a biomedical investigation of the causes and consequences of food allergy. Res Involv Engagem. 2016;2(1). 10.1186/s40900-016-0043-x PMC5831893 29507762

[37] Denzin NK, Lincoln YS, editors. The Sage handbook of qualitative research. 5th ed. United Kingdom: SAGE Publications, Inc,; 2017. ISBN (electronic): 9781506365442.

[38] Hyett N, Kenny A, Dickson-Swift V (2014). Methodology or method? A critical review of qualitative case study reports. Int J Qual Stud Health Well-being.

[39] Yin RK. Applications of case study research. In: Applied Social Research Series. London: Sage; 1993.

[40] Sibbald SL, Paciocco S, Fournie M, Van Asseldonk R, Scurr T (2021). Continuing to enhance the quality of case study methodology in health services research. Healthc Manage Forum.

[41] Stake RE (1995). The art of case study research.

[42] Merriam SB (2009). Qualitative research: a guide to design and implementation.

[43] Tong A, Sainsbury P, Craig J (2007). Consolidated criteria for reporting qualitative research (COREQ): a 32-item checklist for interviews and focus groups. Int J Qual Health Care.

[44] Bradley EH, Curry LA, Devers KJ (2007). Qualitative data analysis for health services research: developing taxonomy, themes, and theory. Health Serv Res.

[45] Robert E, Samb OM, Marchal B, Ridde V (2017). Building a middle-range theory of free public healthcare seeking in sub-Saharan Africa: a realist review. Health Policy Plan.

[46] Srivastava P, Hopwood N (2009). A practical iterative framework for qualitative data analysis. Int J Qual Methods.

[47] Morgan DL, Nica A (2020). Iterative thematic inquiry: a new method for analyzing qualitative data. Int J Qual Methods.

[48] Crosschild C, Huynh N, De Sousa I, Bawafaa E, Brown H. Where is critical analysis of power and positionality in knowledge translation? Health Res Policy Syst. 2021;19:92:1–9. 10.1186/s12961-021-00726-w PMC8196505 34116685

[49] Jull J, Giles A, Graham ID (2017). Community-based participatory research and integrated knowledge translation: advancing the co-creation of knowledge. Implement Sci.

[50] Castleden H, Morgan VS, Lamb C (2012). “I spent the first year drinking tea”: exploring Canadian university researchers’ perspectives on community-based participatory research involving Indigenous peoples. The Canadian Geographer / Le Géographe canadien.

[51] Field B, Booth A, Ilott I, Gerrish K (2014). Using the Knowledge to Action Framework in practice: a citation analysis and systematic review. Implement Sci.

[52] Graham ID, Logan J, Harrison MB, Straus SE, Tetroe J, Caswell W (2006). Lost in knowledge translation: time for a map?. J Contin Educ Heal Prof.

[53] Jagosh J, Macaulay AC, Pluye P, Salsberg J, Bush PL, Henderson J, et al. Uncovering the benefits of participatory research: implications of a realist review for health research and practice. Milbank Quarterly. 2012;90(2):311–46. 10.1111/j.1468-0009.2012.00665.x PMC3460206 22709390

[54] Rycroft-Malone J, Burton CR, Wilkinson J, Harvey G, McCormack B, Baker R (2015). Collective action for implementation: a realist evaluation of organisational collaboration in healthcare. Implement Sci.

[55] Hellström T (2018). Centres of excellence and capacity building: from strategy to impact. Sci Public Policy.

